# Atoms-In-Molecules’ Faces of Chemical Hardness by Conceptual Density Functional Theory

**DOI:** 10.3390/molecules27248825

**Published:** 2022-12-12

**Authors:** Savas Kaya, Mihai V. Putz

**Affiliations:** 1 Department of Pharmacy, Health Services Vocational School, Sivas Cumhuriyet University, Sivas 58140, Turkey; 2 Laboratory of Computational and Structural Physical-Chemistry for Nanosciences and QSAR, Biology-Chemistry Department, Faculty of Chemistry, Biology, Geography, West University of Timișoara, Pestalozzi Str. No. 16A, RO-300115 Timișoara, Romania; 3 Scientific Laboratory of Renewable Energies-Photovoltaics, R&D National Institute for Electrochemistry and Condensed Matter (INCEMC-Timisoara), Dr. Aurel Podeanu Str. No. 144, RO-300569 Timișoara, Romania

**Keywords:** chemical hardness, chemical reactivity principles, conceptual density functional theory, solid-state chemistry

## Abstract

The chemical hardness concept and its realization within the conceptual density functional theory is approached with innovative perspectives, such as the electronegativity and hardness equalization of atoms in molecules connected with the softness kernel, in order to examine the structure–reactivity equalization ansatz between the electronic sharing index and the charge transfer either in the additive or geometrical mean picture of bonding. On the other hand, the maximum hardness principle presents a relation with the chemical stability of the hardness concept. In light of the inverse relation between hardness and polarizability, the minimum polarizability principle has been proposed. Additionally, this review includes important applications of the chemical hardness concept to solid-state chemistry. The mentioned applications support the validity of the electronic structure principles regarding chemical hardness and polarizability in solid-state chemistry.

## 1. Introduction

Since the reformulation of the electronegativity (χ) concept within density functional theory (DFT) [1], it has acquired a central place in chemical reactivity due to its special relationship with the chemical potential (μ = −χ) on the one hand and by its functional definition of the so-called local electronegativity on the other hand, viewed as the functional variation of the density functional energy of a system E[ρ] with respect to the electronic density in a given potential environment [2]:


(1) $$\chi \left(r\right)=-{\left(\frac{\delta \;E[\rho ]}{\delta \rho \left(r\right)}\right)}_{V\left(r\right)}$$

Then, at the global level, electronegativity is written as

(2) $$\chi =\int \chi \left(r\right)f\left(r\right)dr=-\int {\left(\frac{\delta \;E[\rho ]}{\delta \rho \left(r\right)}\right)}_{V\left(r\right)}{\left(\frac{\partial \rho \left(r\right)}{\partial N}\right)}_{V\left(r\right)}dr\;=-\int {\left(\frac{\delta \;E[\rho ]}{\delta \rho \left(r\right)}\right)}_{V\left(r\right)}{\left(\frac{\partial \rho \left(r\right)}{\partial N}\right)}_{V\left(r\right)}dr=-{\left(\frac{\partial E}{\partial N}\right)}_{V\left(r\right)}$$

while recognizing the involvement of the basic DFT local-to-global assumption relating the electronic density conversion to the total number of electrons [3],

(3) ∫ρ(r)dr=N

and the frontier reactivity site driving the Fukui function [4,5]

(4) $${\left(\frac{\partial \rho (r)}{\partial N}\right)}_{V\left(r\right)}=f(r)$$

There is therefore evidence that electronegativity, although having an essential global effect (see Equation (2), contains the local causes related to the electronic density variations Equation (1). These equivalent two sides of the electronegativity concept allow its major involvement in modeling chemical reactivity in general, as well as trigger it [6]. Equally, the consecrated electronegativity companion known as chemical harness may also be formulated through the hierarchical bilocal to local to global series [7,8,9,10]:

(5) $$\begin{array}{l} \eta =\iint \eta \left(r,r^{\prime }\right)f\left(r\right)f\left(r^{\prime }\right)drdr^{\prime }\\ =\frac{1}{2}\iint {\left(\frac{\delta^{2}E[\rho ]}{\delta \rho (r)\delta \rho (r^{\prime })}\right)}_{V\left(r\right)}{\left(\frac{\partial \rho \left(r\right)}{\partial N}\right)}_{V\left(r\right)}{\left(\frac{\partial \rho \left(r^{\prime }\right)}{\partial N}\right)}_{V\left(r\right)}drdr^{\prime } \end{array}\;=\int \eta \left(r\right)f\left(r\right)dr=-\frac{1}{2}\int {\left(\frac{\delta \chi }{\delta \rho (r)}\right)}_{V\left(r\right)}{\left(\frac{\partial \rho \left(r\right)}{\partial N}\right)}_{V\left(r\right)}dr\;=-\frac{1}{2}{\left(\frac{\partial \chi }{\partial N}\right)}_{V\left(r\right)}=\frac{1}{2}{\left(\frac{\partial^{2}E}{\partial N^{2}}\right)}_{V\left(r\right)}$$

with the help of a chemical hardness kernel [11]

(6) $$\eta \left(r,r^{\prime }\right)=\frac{1}{2}{\left(\frac{\delta^{2}E[\rho ]}{\delta \rho (r)\delta \rho (r^{\prime })}\right)}_{V\left(r\right)}$$

and the associated local chemical hardness [12]:

(7) $$\eta \left(r\right)=-\frac{1}{2}{\left(\frac{\delta \chi }{\delta \rho (r)}\right)}_{V\left(r\right)}$$

However, the above procedure may be applied to develop a reactive energy dependency on the chemical indices of electronegativity and chemical hardness through the second-order expansion in terms of electronic density variation, namely

(8) $$E[\rho +\delta \rho ]≅E[\rho ]+\int \int {\left(\frac{\delta \;E[\rho ]}{\delta \;\rho (r)}\right)}_{V(r)}{\left(\frac{\partial \rho (r)}{\partial N}\right)}_{V\left(r\right)}dNdr\;+\frac{1}{2}\iint \iint {\left(\frac{\delta^{2}\;E[\rho ]}{\delta \;\rho (r)\delta \;\rho (r^{\prime })}\right)}_{V}{\left(\frac{\partial \rho (r)}{\partial N}\right)}_{V\left(r\right)}{\left(\frac{\partial \rho (r^{\prime })}{\partial N}\right)}_{V\left(r^{\prime }\right)}dNdNdrdr^{\prime }\;=E[\rho ]+\left[\int {\left(\frac{\delta \;E[\rho ]}{\delta \;\rho (r)}\right)}_{V(r)}f\left(r\right)dr\right]\times \Delta N\;+\frac{1}{2}\left[\iint {\left(\frac{\delta^{2}\;E[\rho ]}{\delta \;\rho (r)\delta \;\rho (r^{\prime })}\right)}_{V}f\left(r\right)f\left(r^{\prime }\right)drdr^{\prime }\right]\times {\left(\Delta N\right)}^{2}\;=E[\rho ]-\chi \Delta N+\frac{1}{2}\eta {\left(\Delta N\right)}^{2}$$

**Figure 1:**
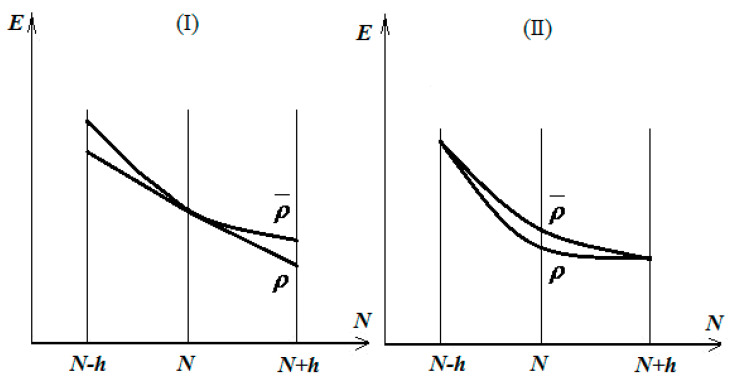
The two cases of the total energy minimization by distinctly acting on the parabolic energetic shape connecting the electronic states |N−h〉,  |N〉  , and |N+h〉 ; see the text for details.

Then, one is interested in minimizing the energy throughout all parabolic classes that link those states. This minimizing procedure can be undertaken immediately in two distinct ways: by simultaneously minimizing the energetic values of the states |N−h〉 and  |N+h〉 or by only acting on the energy in the “point”  |N〉  of the energetic shape, as in cases I and II in Figure 1, respectively [13,14].

Firstly, in both analyzed cases in Figure 1, the electronegativity approaches its minimum on the right ground density *ρ*, around the electronic state  |N〉 

(9) $$\left(I)\;\;,\;\;(II)\;\;:\;\;\bar{\chi }\;\;[\bar{\rho }]\;\;\geq \;\;\chi \;\;[\rho \right]$$

whereas the chemical hardness records correspond with case II; otherwise, the minimization of energy tends to deform the parabolic energetic shape into a linear one, thus prescribing the maximum hardness optimization for the achievement of the right ground state, i.e., the maximum hardness principle for the equilibrium of the many electronic systems in their ground states [15].

In particular, although various electronic regions (basins) bind within a stable molecule, they minimize their electronegativity difference by equalizing their associated chemical potentials so that the variation principle (9) is rewritten as the limit (10) [16,17,18,19,20]:

(10) Δχ→min→0

Conversely, chemical reactivity is promoted by non-zero, big Δχ, and is qualitatively expressed by the principle of frontier reactivity: out of two different sites with a generally similar disposition for reacting with a given reagent, the reagent prefers the one on the reagent’s approach that is associated with the maximum response of the system’s electronegativity [21,22]. 

In this context, the present paper explores the possible connections between the local behavior of atomic basins through the electronic density and associated quantities and the observed electronegativity effect on bonding as a global measure of reactivity that preludes bonding.

## 2. Chemical Hardness-Softness Driving Chemical Bonding

### 2.1. The Electonic Sharing Ansatz 

Modeling the chemical bonding by atoms in the molecule paradigm has spanned almost 100 years of cornerstone approaches, starting from the “cubic atom” of Lewis [23] and continuing with Langmuir’s pre-quantum approach [24]; the seminal works of Thomson [25]; Heitler and London’s foundations of molecular orbital theory [26]; Hückel [27]; Pauling [28]; Roothann’s atomic orbitals’ linear combinations in the molecule [29], the celebrated Pariser–Parr–Pople semiempirical approach to π-systems [30,31,32], the formalization of the Hartree–Fock method in the context of density matrices [33,34,35], and eventually the density functional theory [36,37,38]. 

It is particularly rewarding that all notions developed so far are currently incorporated into what became the conceptual density functional theory [39,40], with considerably enriched possibilities for analytical and computational developments while considerably expanding the range of applications from small molecules to nano- and bio-electronic systems [41,42,43,44,45]

However, the special inter-electron interaction, either under exchange or correlation formulations, has attracted much attention for properly describing many electronic effects [46,47,48]. In particular, when learning from the phenomenological bonding picture of atoms in molecules provided in Bader’s theory [49,50,51], one should go further by characterizing the amount of electronic sharing between two atoms (A,B) in the molecule or bonding AB using the so-called electronic covariance D(A,B) between the electron atomic populations of A and B [52,53]:

(11) $$D(A,B)=2\int_{A}\int_{B}\rho_{XC}\left(r,r^{\prime }\right)dr^{\prime }dr$$

written with the help of the exchange–correlation density

(12) $$\rho_{XC}\left(r,r^{\prime }\right)=\rho \left(r\right)\rho \left(r^{\prime }\right)-\rho_{2}\left(r,r^{\prime }\right)$$

with the remarkable property it integrates into the total number of electrons of each isolated system

(13) $$\int_{A}\int_{A}\rho_{XC}\left(r,r^{\prime }\right)dr^{\prime }dr=N_{A}$$

because of the second-order density matrix $\rho_{2}\left(r,r^{\prime }\right)$ it integrates in a specific Löwdin manner:

(14) $$\int_{A}\int_{A}\rho_{2}\left(r,r^{\prime }\right)dr^{\prime }dr=N_{A}\left(N_{A}-1\right)$$

whereas the simple density is integrated as usual into DFT (see Equation (3) above).

Although the quantity (11) was not designed to have the predictive power for assessing how the electron sharing can change upon external perturbation, it may be related to the charge transfer of electrons between the two binding regions A and B compared to Equation (13), which refers to isolated regions. This may advance the delocalization of the electronic ansatz

(15) $$\frac{1}{2}D(A,B)\equiv \Delta N_{AB}$$

that gives an exchange–correlation representation for the electronic transfer when triggering the bonding formation. It may be also seen as the reactive version (or a variation) of the structural counterpart of Equation (3). 

Such a connection between the local exchange–correlation and globally recorded effects in bonding may be confirmed when further chemical reactivity indices are expressed in terms of the exchange–correlation density, therefore involving the electronic covariance quantity above (Equation (6)). This is the case for the recently proposed softness kernel [8,54]:

(16) $$s\left(r,r^{\prime }\right)=-{\left(\frac{\partial \rho_{XC}\left(r,r^{\prime }\right)}{\partial \chi }\right)}_{V(r)}$$

which is integrated to produce the global softness of a given region [55]:

(17) $$\begin{array}{l} \iint s\left(r,r^{\prime }\right)drdr^{\prime }=-\int {\left(\frac{\partial }{\partial \chi }\int \rho_{XC}\left(r,r^{\prime }\right)dr^{\prime }\right)}_{V(r)}dr\\ =-\int {\left(\frac{\partial \rho \left(r\right)}{\partial \chi }\right)}_{V(r)}dr=\int s(r)dr=S \end{array}$$

or the corresponding softness covariance S(A,B) when integration into two atomic regions is performed:

(18) $$\int_{A}\int_{B}s\left(r,r^{\prime }\right)drdr^{\prime }=-{\left(\frac{\partial }{\partial \chi }\int_{A}\int_{B}\rho_{XC}\left(r,r^{\prime }\right)drdr^{\prime }\right)}_{V(r)}=-\frac{1}{2}{\left(\frac{\partial D(A,B)}{\partial \chi_{AB}}\right)}_{V(r)}$$

Now, by comparing the isolated and binding (covariance) forms of the actual softness kernel integration in Equations (17) and (18), it appears that the latter should be represented by the former, i.e.,

(19) $$-\frac{1}{2}{\left(\frac{\partial D(A,B)}{\partial \chi_{AB}}\right)}_{A}\equiv S_{B}$$

in the same hierarchical manner that Equations (13) and (3) advance Equation (15) via Equation (11). In other words, if one assumes Equation (15) to be true, the relation (19) should also be fulfilled since it represents the same conceptual ordering that connects the reactive charge-transfer causes (on the right) with the electronic delocalization effects in the formed bond (on the left). If true, such a connection will lead to the continuous description of bonding, from the reactivity (isolated indices) to the bond (shared effects). The first conceptual test of such an ansatz is in the next exposure referring to the electronegativity equalization of atoms in molecules treated in various bonding contexts. 

### 2.2. Additive Equalization of Atoms in Molecules

Here we will treat the so-called Parr–Pearson–Nalewajski additive model of atoms in molecules [56,57]. Let us consider the formation of a diatomic molecule AB or a bond with constant atomic nuclear charges at the equilibrium separating distance $R_{AB}$. For an infinitesimal transfer of electronic charges between the isolated atoms (A, B) to achieve their atoms in molecules states (〈A〉, 〈B〉)

(20) $$N_{\langle A\rangle }=N_{A}-dN_{\langle A\rangle }$$

(21) $$N_{\langle B\rangle }=N_{B}-dN_{\langle B\rangle }$$

the variation in the total energy

(22) $$E=E_{\langle A\rangle }+E_{\langle B\rangle }$$

can be written as

(23) $$dE={\left(\frac{\partial E}{\partial \;N_{\langle A\rangle }}\right)}_{N_{\langle B\rangle },\;R_{AB}}(N_{\langle A\rangle }-N_{A})-{\left(\frac{\partial E}{\partial \;N_{\langle B\rangle }}\right)}_{N_{\langle A\rangle },\;R_{AB}}(N_{\langle B\rangle }-N_{B})\;+{\left(\frac{\partial E}{\partial R_{AB}}\right)}_{N_{\langle A\rangle },\;N_{\langle B\rangle }}dR_{AB}$$

Since in the fundamental equilibrium state

(24) $$\left\{ \begin{array}{l} dE=0\\ \frac{\partial E}{\partial R_{AB}}=0 \end{array}\right.$$

Equation (22) is simplified to

(25) $${\left(\frac{\partial E_{\langle A\rangle }}{\partial N_{\langle A\rangle }}\right)}_{N_{\langle B\rangle },\;R_{AB}}={\left(\frac{\partial E_{\langle B\rangle }}{\partial N_{\langle B\rangle }}\right)}_{N_{\langle A\rangle },\;R_{AB}}$$

which recovers in an analytical form the principle (10) of the equalization of the atoms in molecules’ electronegativities. Next, by unfolding this principle one first yields

(26) $$\chi_{\langle A\rangle }=\chi_{A}-2\eta_{A}\Delta N=\chi_{\langle B\rangle }=\chi_{B}+2\eta_{B}\Delta N=\chi_{AB}$$

which provides the finite amount of the transferred charge:

(27) $$\Delta N=\frac{\chi_{A}-\chi_{B}}{2(\eta_{A}+\eta_{B})}$$

and the associated equalized electronegativity of atoms in molecules,

(28) $$\chi_{AB}=\chi_{\langle A\rangle }=\chi_{\langle B\rangle }=\frac{\chi_{A}\eta_{B}+\chi_{B}\eta_{A}}{2\left(\eta_{A}+\eta_{B}\right)}$$

Consequently, with the help of parabolic energy, Equation (8) adapted to the present atoms in molecules viewed as a perturbed state respecting that of the isolated atoms, the bonding “working” energy successively becomes

(29a) $$\Delta E=(E_{\langle A\rangle }-E_{A})+(E_{\langle B\rangle }-E_{B})=\left(-\chi_{A}+\eta_{A}\Delta N+\chi_{B}+\eta_{B}\Delta N\right)\Delta N\;=-\frac{1}{4}\frac{{\left(\chi_{A}-\chi_{B}\right)}^{2}}{\left(\eta_{A}+\eta_{B}\right)}$$

Now, it is clear that the electronegativity difference is crucial for binding promotion and bonding formation, as well as in terms of the exchanged number of electrons for the total energy released. 

At this point, one may check that the ansatz (15) under the present form

(29b) $$\frac{1}{2}D\left(A,B\right)=\frac{\chi_{A}-\chi_{B}}{2(\eta_{A}+\eta_{B})}$$

leads with a softness-type Equation (19):

(30a) $$\begin{array}{l} -\frac{1}{2}{\left(\frac{\partial D(A,B)}{\partial \chi_{AB}}\right)}_{A}=-\left(\frac{1}{2}\frac{\partial D(A,B)}{\partial \chi_{A}}\right)\left(\frac{\partial \chi_{A}}{\partial \chi_{AB}}\right)\\ =-\frac{1}{2(\eta_{A}+\eta_{B})}\frac{2(\eta_{A}+\eta_{B})}{\eta_{B}}=-\frac{1}{\eta_{B}}\equiv -2S_{B} \end{array}$$

(30b) $$-\frac{1}{2}{\left(\frac{\partial D(A,B)}{\partial \chi_{AB}}\right)}_{B}=+\frac{1}{\eta_{A}}\equiv +2S_{A}$$

Therefore, the change in the electron sharing between A and B upon the modification of the chemical potential in A depends on the softness of B. Since the chemical potential is conceptually equivalent to the electronegativity, we reach the intuitive condition that the change in electronegativity in A will bring a change in the electron sharing between A and B, which is proportional to the softness of B. The present example highlights the significance of the new softness kernel (16).

### 2.3. Geometrical Equalization of Atoms in Molecules

Alternatively, one may consider the so-called geometrical mean of the modeling stage of atoms in molecules, namely through the relationships [58]

(31a) $$\chi_{AB}=\chi_{\langle A\rangle }=\chi_{\langle A\rangle }(N_{A}+\Delta N)$$

(31b) $$\chi_{AB}=\chi_{\langle B\rangle }=\chi_{\langle B\rangle }(N_{B}-\Delta N)$$

Together, Equations (31a) and (31b) can equivalently be written as

(32) $$\chi_{AB}^{2}=\chi_{\langle A\rangle }\chi_{\langle B\rangle }=\chi_{\langle A\rangle }(N_{A}+\Delta N)\chi_{\langle B\rangle }(N_{B}-\Delta N)$$

For energy conservation reasons, a similar relationship has to take place in terms of the isolated atoms:

(33) $$\chi_{AB}^{2}=\chi_{A}\chi_{B}$$

By equating the right-hand sides of the results of the last two equations, the geometrical general form for the dependence of the atomic electronegativity in molecules is

(34) $$\chi_{\langle \rangle }=\chi \;\exp[-\gamma \;\Delta N]=\chi \;\exp[-\gamma \;(N_{\langle \rangle }-N)]$$

with

(35) $$\Delta N=\left\{ \begin{array}{l} +\Delta N...forA\\ -\Delta N...forB \end{array}\right.$$

being *γ* an exponential scaling parameter, with the working expression

(36) $$\gamma =\frac{2\eta_{\langle \rangle }}{\chi_{\langle \rangle }}$$

since

(37) $$2\eta_{\langle \rangle }=-\frac{\partial \chi_{\langle \rangle }}{\partial N_{\langle \rangle }}\;=\gamma \chi \exp[-\gamma \;\;\Delta N]\;=\gamma \chi_{\langle \rangle }$$

Under these conditions, by applying the equalization principle to the atomic electronegativities in molecules,

(38) $$\chi_{\langle A\rangle }=\chi_{\langle B\rangle }$$

the charge transferred within the binding process of the AB molecule is found:

(39) $$\Delta N=-\frac{1}{2\gamma }\ln\left(\frac{\chi_{B}}{\chi_{A}}\right)$$

For completeness, the corresponding released energy will be calculated now as

(40) $$-\Delta E=\int_{N_{A}=Z_{A}^{*}}^{N_{A}+\Delta N}\chi_{\langle A\rangle }dN_{\langle \rangle }+\int_{N_{B}=Z_{B}^{*}}^{N_{B}-\Delta N}\chi_{\langle B\rangle }dN_{\langle \rangle }\;=\chi_{A}\frac{1}{\gamma }\;\;[1-\exp(-\gamma \;\Delta N)]+\chi_{B}\frac{1}{\gamma }\;\;[1-\exp(+\gamma \;\Delta N)]\;≅-(\chi_{B}-\chi_{A})\Delta N-\frac{1}{2}(\chi_{A}+\chi_{B})\gamma \;\;{\left(\Delta N\right)}^{2}$$

As can be seen, the geometrical mean in Equation (39) is richer than the additive model in Equation (27) for the atoms in molecules’ charging transfer since it also contains the equilibrium information through the parameter of Equation (36). Consequently, it can be assumed for the electronic sharing index of atoms in molecules through Equation (15)

(41) $$\frac{1}{2}D(A,B)\equiv \Delta N=\frac{1}{2\gamma }\ln\left(\frac{\chi_{A}}{\chi_{B}}\right)$$

leading with the successive softness-type relationships

(42a) $$-\frac{1}{2}{\left[\frac{D(A,B)}{\partial \chi_{\langle \rangle }}\right]}_{A}=-\frac{1}{2}\left[\frac{\partial D(A,B)}{\partial \chi_{A}}\right]\left(\frac{\partial \chi_{A}}{\partial \chi_{\langle \rangle }}\right)\;=-\frac{1}{2\gamma \chi_{A}\exp[-\gamma \;\Delta N]}=-\frac{1}{2\gamma \chi_{\langle \rangle }}=-\frac{1}{4\eta_{\langle \rangle }}=-\frac{1}{2}S_{\langle \rangle }$$

and, in a similar way,

(42b) $$-\frac{1}{2}{\left[\frac{D(A,B)}{\partial \chi_{\langle \rangle }}\right]}_{B}=+\frac{1}{2}S_{\langle \rangle }$$

being however a quarter of the numerical electronic sharing indices specific to the additive model of atoms in molecules compared with Equations (30a) and (30b), therefore modeling weaker bonds. Moreover, the geometrical mean model prescribes that the sharing index behaves like the bonding softness at the atoms-in-molecules level, unlike the additive model that ends with the softnesses of isolated atoms; it is, therefore, more adapted to the binding reality being consistent with the molecular realm either in its structure (sharing) index or its reactivity counterpart (i.e., chemical softness).

## 3. Applications of Chemical Hardness in Solid-State Chemistry 

Chemical hardness is reported as the resistance against the polarization of the electron cloud of atoms, ions, and molecules [59]. Following the hard and soft acid-base (HSAB) principle, Pearson proposed the maximum hardness principle (MHP). According to the MHP [60], “There seems to be a rule of nature that molecules arrange themselves so as to be as hard as possible.” It can be deduced from this explanation that chemical hardness is an indicator of chemical stability. Many studies published have proved that hard chemical systems are more stable than soft ones. Another electronic structure rule based on developments in conceptual density functional theory is the minimum polarizability principle (MPP) of Chattaraj [61]. The MPP implies the minimization of polarizability in stable states and conformations. 

On the other hand, lattice energy (U) is one of the most popular parameters in solid-state chemistry. This parameter is defined as the energy required to decompose the gaseous ions in the solids. Many famous equations derived for the prediction of the lattice energies of organic and inorganic ionic systems are available, especially in inorganic chemistry textbooks. Some of these equations are the Born–Lande, Born–Mayer, and Kapustinskii equations. Among these equations, only the Kapustinskii equation can also be used for the prediction of lattice energies of inorganic ionic crystals of unknown lattice types. This equation derived by Kapustinskii is given as [62]

(43) $$U=\frac{A\left|\nu z_{+}z_{-}\right|}{\langle r\rangle }\left(1-\frac{\rho }{\langle r\rangle }\right)$$

Here, z+ and z− are the charges on the cation and anion of the crystal, respectively. ν is the number of ions in the formula unit of the considered ionic crystal. ρ is reported as a compressibility constant with a numerical value of ρ = 0.0345 nm. <r> represents the sum of the ionic or thermochemical radii of the ions in the crystal.

In 2015, Kaya and Kaya [63] derived a new equation to compute the hardness of molecules in light of the hardness equalization principle. According to the hardness equalization principle of Datta, the hardnesses of atoms during molecule formation become equalized like their electronegativities. It is important to note that some important criticisms of the hardness equalization principle were made by Prof Laszlo von Szentpaly [64]. The molecular hardness (η_M_) equation derived by Kaya and Kaya is given as

(44) $$\eta_{M}=\left[(2\sum_{i=1}^{N}b_{i}/a_{i})+q_{M}\right]/\left[\sum_{i=1}^{N}1/a_{i}\right]$$

In the given equation, *N* represents the number of atoms in the molecule. *q_M_* is the charge of the molecule. In particular, this equation can be used for both neutral and charged molecular systems. *a_i_* and *b_i_* are the parameters depending on the ground-state ionization energy (*I*) and electron affinity (*A*) of any atom i in the molecule and these parameters are calculated as

(45) $$a_{i}=\frac{I_{i}+A_{i}}{2}$$

(46) $$b_{i}=\frac{I_{i}-A_{i}}{2}$$

The inverse relation between hardness and polarizability was highlighted by Ghanty and Ghosh [65]. The authors noted the proportionality between the cube root of the polarizability (α) and the softness (multiplicative inverse of the hardness). Using the inverse relation between hardness and polarizability, Chattaraj and Sengupta proposed the minimum polarizability principle (MPP), stating that “in the stable states, polarizability is minimized”. A long time before the introduction of the MPP, Prof. Jenkins and Prof. Glasser introduced the volume-based thermodynamics approach (VBTA) as a result of many quality papers published in the literature [66]. Although the authors (Prof. Jenkins and Prof. Glasser) who introduced the volume-based thermodynamics approach did not mention it in their published papers, the success of VBTA in solid-state chemistry is easily explained through the MPP and MHP. The VBT approach calculates important properties, such as the lattice energy, standard absolute entropy, surface tension, and heat capacity of organic and inorganic systems, using only their molar volumes [67]. The proportionality between the molar volume and the polarizability has been given via the Lorentz–Lorenz equation. 

One of the most important studies on the use of the concept of molar volume in solid-state chemistry was introduced by Bartlett in the 1980s [68]. Limited solid-state calculations are possible with the help of the Bartlett relation given by Equation (47). These limited solid-state chemistry calculations based on the use of molar volume in the Bartlett relation were replaced by very popular equations thanks to the volume-based thermodynamics approach of Jenkins and Glasser [69]. It is important to note that the Bartlett relation showing the relation between the lattice enthalpy (*ΔH_L_*) and molecular volume (*V_m_*) has been presented for simple MX (1:1) salts.

(47) $$\Delta H_{L}(\mathrm{kJ}/\mathrm{mol})=\frac{232.8}{V_{m}{}^{1/3}}+110$$

The lattice-energy equation derived in the scope of the VBT approach is

(48) $$U(kJ/mol)=2I\left[\alpha \frac{1}{V_{m}{}^{1/3}}+\beta \right]$$

where α and β are the constants based on the stoichiometry of the inorganic ionic crystal. *I* stands for the ionic strength and it is calculated for a crystal as

(49) $$I=1/2\sum n_{i}z_{i}{}^{2}$$

where n_i_ is the number of ions and z_i_ is the charge of the ion. 

Investigating the link between chemical hardness (η) and lattice energy, a few years ago, Kaya and Kaya [70] derived a new lattice-energy equation based on the chemical hardness concept. This equation is presented as

(50) $$U,\;kJ/mol=2I\left[a\frac{\eta_{M}}{V_{m}{}^{1/3}}+b\right]$$

where a and b are stoichiometry-dependent constants. η_M_ is the molecular hardness. Another electronic structure principle proposed for the prediction of the exothermic and endothermic nature of chemical reactions is the minimum electrophilicity principle. This principle, which was proposed by Chattaraj, Chamorro, and Fuentealba [71], states that in an exothermic reaction, the sum of the electrophilicity indexes of products should be smaller than that of the reactants. In a recent paper, Szentpaly and Kaya [72] reanalyzed the validity and limitations of the minimum electrophilicity principle via some solid-state double-exchange reactions. In the same paper, the authors introduced the maximum composite hardness rule. According to this rule, the exothermic and endothermic nature of solid-state double-exchange reactions can be predicted from the following relations

(51) $$\Delta \eta /V^{1/3}>0\;(\mathrm{exothermic})$$

(52) $$\Delta \eta /V^{1/3}<0\;(\mathrm{endothermic})$$

For a reaction, Δη/V_m_^1/3^ is calculated as

(53) $$\Delta \eta /V^{1/3}=\sum_{i=1}^{n}(\eta /V^{1/3}{}_{product}{)}_{i}-\sum_{j=1}^{m}(\eta /V^{1/3}{}_{reac\tan t}{)}_{j}$$

where *m* and *n* represent the number of reactants and the number of products, respectively.

Carlos Cardenas [73] reported that the Fukui potential can be considered a measure of the chemical hardness of atoms. The Fukui potential (υ f(r)) is known as the electrostatic potential due to a distribution of charge equal to the Fukui function, f(r). For the calculation of the Fukui potential, the following equations are used [74]:

(54) $$\upsilon_{f}^{+}(r)=\int \frac{f^{+}(r^{\prime })}{\left|r-r^{\prime }\right|}dr^{\prime }$$

(55) $$\upsilon_{f}^{-}(r)=\int \frac{f^{-}(r^{\prime })}{\left|r-r^{\prime }\right|}dr^{\prime }$$

where, + and − stand for the gaining of electrons and the removal of electrons, respectively.

Because chemical hardness is strongly related to the stability of the chemical system, the relation highlighted by Cardenas between the Fukui potential and chemical hardness implies that the Fukui potential can also be used in solid-state chemistry calculations such as chemical hardness. 

In a recent paper, Kaya, Gomez, and Cardenas [75] derived a new formula based on the Fukui potential for the prediction of lattice energies of inorganic ionic systems. The mentioned formula is given as

(56) $$U=g{\left(v^{-}{\left(R_{metal}\right)}^{m}v^{+}{\left(R_{non-metal}\right)}^{n}\right)}^{1/(m+n)}+j$$

In the given formula, g and j are the constants taking different numerical values for different stoichiometries. *m* and *n* are the numbers of metals and non-metals in the structure. 

## 4. Bond Force Constant and Chemical Hardness

The experimentally direct measuring of the bond force constant of molecules is not possible. For this reason, in the literature, many theoretical methodologies have been suggested to compute the bond force constants of molecules. One of the most well-known models is Badger’s bond force constant equation, which is given below [76]:

(57) $$k=\frac{1.8\times 10^{5}}{{\left(r_{e}-d_{ij}\right)}^{3}}$$

where k is the bond-stretching force constant in dyn cm^−^^1^ units. r_e_ represents the internuclear distance in Angstrom units and d_ij_ is a function of the position of the bonded atoms in the periodic table.

Pearson [77], who introduced the chemical hardness concept, proposed the following equation (Equation (58)) to compute the bond force constant of AB-type diatomic molecules. Then, Nalewajski, noticing some deficiencies in the bond force constant equation of Pearson, derived a new bond force constant equation (Equation (59)) [78]:

(58) $$k_{AB}=2\frac{Z_{A}Z_{B}}{R_{0^{3}}}$$

(59) $$k_{AB}=(Z_{A}\cdot \delta_{A})(Z_{B}\cdot \delta_{B})2^{n_{A}+n_{B}-2}R_{e}{}^{3}$$

where *Z_A_*, and *Z_B_*, are the effective charges on the A and B atoms, respectively. *R_0_* is the equilibrium nuclear separation. Nalewajski proposed the $z_{X}=(Z_{X}+\delta_{X})/2^{n_{X}-1/2}$ equation to calculate the effective charge (*z_x_*) in terms of the location of atom X in the periodic table. Here, Z_X_ stands for the atomic number. For an atom X from groups IA-VA of the periodic table, *δ_X_* is equal to zero. δ_X_ for any X atom from groups VIA and VIIA is calculated as $\delta_{X}=5-\upsilon_{X}$. Re represents the bond length. *n_X_* and *υ_X_* are the group and period of atom X, respectively. 

It is well known that bond force constants of molecules help us to comment on their stability. From this explanation and the maximum hardness principle, it is not difficult to relate the bond force constant to the chemical hardness concept. Firstly, Kaya and coworkers [79] presented the following chemical hardness-based equation for the prediction of the bond force constants of diatomic molecules:

(60) $$k_{AB}=c_{1}\frac{\eta_{M}}{R_{e}{}^{2}}+c_{2}$$

where *k_AB_* is the bond force constant in [N/m] units and η_M_ is the molecular hardness in [eV] units. Re represents the bond length of the diatomic molecule. *c_1_* and *c_2_* are constants with different numerical values for different molecule groups. Another molecular hardness-based bond force constant equation is given as [80]

(61) $$k=p\frac{\eta_{M}}{V_{m}{}^{1/3}}+q$$

In the given equation, *V_m_* is the molar volume of the inorganic ionic compound. *p* and *q* are constants and their numerical values for alkali halides are 11.278 and −60.541, respectively. 

## 5. Conclusions

The “battle” for elucidating the nature of the (quantum) chemical bond [81,82,83,84,85,86] is fundamental to establishing the future of newly directed chemical synthesis with predefined properties and reactivity aiming at specific interactions (with cutting-edge use in designing new compounds with an active role in health, food, and the environment at large). The chemical bonding peculiarities are rooted in the interelectronic interaction in the confined molecular space, which requires “limiting physics” since the chemical bond essentially results in the attraction (and, in any case, in a relatively stable) of two or many charges with the same sign (electrons), which otherwise should be repulsive in “free motion physics”. The key to this apparent “chemical bonding paradox” lies in the mixed action at both the energy and wave-function or density (not only conceptual) levels on the one hand—since they introduce an arsenal including dynamical partition, invariance, conservation, virial, exchange, kinetics, correlation, and other operator-*energy* related quantities—and the superposition, tunneling, interference, collapses, collectivization, bosonization, superfluidization, and even entangling [87] on the electronic *wave-function or density* behavior on the other hand [88,89]. This energy–wave-function/density mix is quantum-mechanically established since they both act as eigenvalues and eigenfunctions of the same Schrödinger or Hartree–Fock equations, with all related variants on one side and the energy and electronic density coupling within density functional theory on the other side. Therefore, the energy–wave-function/density mix is naturally provided in the “one-shot/equally footing” output within the inner dynamics, as well as in the eventually observable chemical characteristics of the electronic systems as with atoms in molecules [81,86,88,89]. Indeed, density alone—even complemented by its Laplacean, for instance—as in the “purist” Bader’s theory of bonding (critical) paths [82,85], does not suffice for correctly predicting the observable energy-related chemical features or their reactivity [83,84]. So, in order to have a comprehensible picture of (the causes of) observable chemical phenomena, energy-related considerations should be called along electronic density shapes since they both are quantum mechanical quantities (describing the structural rearrangements, pairings, accumulations, charge concentrations or depletions, or fragmentation realities) linked in the eigenequations of electronic–field interactions (including the Pauli repulsion, electrostatic interaction, orbital/superposable interaction, exchange and correlation, and even fermionic fields’ bosonization [90], etc.). Accordingly, the “observable” forms of the related chemical reactivity indices, as are those rooted in electronegativity [91] as the first-order energy–density relationship, while leaving open the question of the observability of the second order of the energy–density relationship—as it is the chemical hardness [92]—are the main subjects of the present paper in various dynamical contexts. This way, the intriguing issue of making the contact between the chemical structure and chemical reactivity was approached using the electronic sharing (electronic covariance) in bonds and the electronegativity of atoms in molecules, respectively. The main findings can be summarized as follows:

- The chemical information contained within the basic density functional of the total number of electrons in terms of simple electronic density may also be regarded at the level of the exchange–correlation density, enriching the inter-electronic effects.
- As a consequence, the related electronic covariance in the bond may eventually be equated with the charge transfer in bonding in what is considered the first-order level of the structure–reactivity density functional connection.
- Assuming the previous structure–reactivity connection, the variation in the electronic covariance respecting the equalized electronegativity of atoms in molecules behaves like the softness of the partners in bonding and the entire bonded system for additive and product (geometrical mean) models of atoms in molecules, respectively; it may thus be viewed as the second-order level of the structure–reactivity connection.
- The maximum hardness and minimum polarizability principles have important applications in solid-state chemistry. These applications can be accepted as strong linkages between solid-state chemistry and the conceptual density functional theory.
- The chemical hardness and Fukui potential provide important hints about the stabilities of inorganic ionic systems and these descriptors can be easily used in the calculation of the lattice energies of inorganic crystals.
- The chemical hardness and molar volume of inorganic ionic systems can be used in the calculation of their bond force constants.
- The previously questionable observable characteristics of chemical hardness based on the second quantification framework [92] are partly determined here as being positive for the limiting cases of unitary electronic density on the frontier states, i.e., for ionization energy/HOMO and affinity energy/LUMO, or on the valence and conduction levels in the solid-state bands of chemical solids, respectively.

Further works are therefore encouraged and required for further exploration of the implications of the present structure–reactivity relationship for predicting the chemical stability, aromaticity, and computational design of compounds with pre-definite structural or reactivity properties.

## Data Availability

Not applicable.
